# Evolutionary diversification of an ancient gene family (*rhs*) through C-terminal displacement

**DOI:** 10.1186/1471-2164-10-584

**Published:** 2009-12-07

**Authors:** Andrew P Jackson, Gavin H Thomas, Julian Parkhill, Nicholas R Thomson

**Affiliations:** 1 The Wellcome Trust Sanger Institute, Genome Campus, Hinxton, Cambridge, CB10 1SA, UK; 2 Department of Biology, University of York, PO Box 373, York, YO10 5YW, UK

## Abstract

**Background:**

*Rhs *genes are prominent features of bacterial genomes that have previously been implicated in genomic rearrangements in *E. coli*. By comparing *rhs *repertoires across the Enterobacteriaceae, this study provides a robust explanation of *rhs *diversification and evolution, and a mechanistic model of how *rhs *diversity is gained and lost.

**Results:**

*Rhs *genes are ubiquitous and comprise six structurally distinct lineages within the Enterobacteriaceae. There is considerable intergenomic variation in *rhs *repertoire; for instance, in *Salmonella enterica*, *rhs *are restricted to mobile elements, while in *Escherichia coli *one *rhs *lineage has diversified through transposition as older lineages have been deleted. Overall, comparative genomics reveals frequent, independent gene gains and losses, as well as occasional lateral gene transfer, in different genera. Furthermore, we demonstrate that Rhs 'core' domains and variable C-termini are evolutionarily decoupled, and propose that *rhs *diversity is driven by homologous recombination with circular intermediates. Existing C-termini are displaced by laterally acquired alternatives, creating long arrays of dissociated 'tips' that characterize the appearance of *rhs *loci.

**Conclusion:**

*Rhs *repertoires are highly dynamic among Enterobacterial genomes, due to repeated gene gains and losses. In contrast, the primary structures of *Rhs *genes are evolutionarily conserved, indicating that *rhs *sequence diversity is driven, not by rapid mutation, but by the relatively slow evolution of novel core/tip combinations. Hence, we predict that a large pool of dissociated *rhs *C-terminal tips exists episomally and these are potentially transmitted across taxonomic boundaries.

## Background

Enterobacterial genomes are far from stable entities undergoing a constant process of gene acquisition and loss [1]. Genome flux can have a profound effect on the particular organism and in many instances is associated with adaptation to different niches and may eventually come to define different isolates, pathotypes or even species [2]. Genome flux can occur by Lateral Gene Transfer (LGT) though processes such as natural transformation, bacteriophage mediated transduction and conjugation [3-6]. In addition, the expansion of different gene families through gene duplication can introduce functional variation into a population, especially where DNA transfer is restricted. This can act to increase gene dosage or may increase repertoire of genes encoding variable but functionally related proteins within a genome such as the Pmp proteins from *Chlamydophila abortus *[7] and the *Lpl *tandem gene arrays in *Staphylococcus aureus *[8]. Hence, gene gain, gene loss through deletions, genomic rearrangements and the accumulation of point mutations all have important roles in genome flux and have been linked to host adaptation [9-13]

When comparing the architecture of enterobacterial genomes it is clear that they consist of a conserved core inter-dispersed with variable functions that comprise the accessory genome [14]. Studies of *E. coli *have shown that the genomes carry between 4, 238 - 5,589 CDSs (K12 and CFT073; [15]) of which as few as 2,344 CDSs are present in all isolates. Moreover, it is estimated that the *E. coli *pan genome is open and likely to encompass more than 13,000 CDSs [16]. For bacteria living in complex environments it may be assumed that there would be a pressure to continually expand the metabolic and functional flexibility and therefore the genome size. However, there are several factors that are thought to limit genome expansion including the underlying mutation rate, population size and recombination frequency. LGT is also an important factor in preventing the loss of functions that are only weakly beneficial and are normally transferred by vertical inheritance. Consequently LGT within and between species may restore gene function that was previously lost in the recipient, but retained in the donor. Looking at the functions that are represented in the accessory genome of most free-living bacteria the largest single class of genes encode phage or phage-related proteins. In addition there are genes which have been associated with lifestyle or disease outcome, including the LEE pathogenicity island [17] or the high pathogenicity island [18], which are widely distributed amongst the enteric bacteria and whose functions have been intensively studied. However there are other genes that are commonly found within accessory regions in enterobacteria but which are poorly understood; these include the subject of this study, *rearrangement hot-spot*, or *rhs *elements.

*Rhs *elements were first described in *Escherichia coli *K-12 in 1984 using a genetic screen to detect specific amplification of the *glyS *gene. The genetic screen was performed under conditions which selected for the duplication of the *glyS *gene following a *recA*-dependent unequal cross-over event between *rhs *elements *rhsA *and *rhsB*. This explains the *rhs *nomenclature which is derived from these initial descriptions showing that *rhs *elements frequently acted as re-arrangement hot spots in *E. coli *under certain selective conditions [19]. Subsequent work by Charles W. Hill and colleagues identified 5 *rhs *elements in *E. coli *K-12 (*rhsA-E*) as well as additional elements in other members of the ECOR collection [20,21]. Studies looking at the *E. coli rhs *elements showed that they fell into three subfamilies *rhsABCF, rhsDE *and *rhsGH *[21]. These comparisons also revealed that *rhs *loci as defined by Hill and co-workers include a gene composed of an N-terminal G+C rich conserved core region, encoding ~1200 amino acids, and a C-terminal A+T rich region that is highly divergent and was denoted the core-extension (130-177 amino acids). The core protein carries multiple tandemly repeated copies of a YD-repeat domain associated with carbohydrate binding [22,23]. Other genes have also been associated with *rhs *loci including *vgr *and *hcp *genes now known to be associated with Type VI secretion systems [24,25]. However the function of these proteins is unknown and the conditions under which they are expressed have also been difficult to define.

Our knowledge of the distribution of *rhs *elements was initially restricted to *E. coli *although related elements have now been reported in a wide range of organisms including other enterics such as *Salmonella *and *Yersinia *as well as pseudomonads and *Actinobacillus *[26]. A recent paper identified substantial *rhs *genetic polymorphism among strains of a single pathogen (*E. coli *0157:H7) and discussed the use of *rhs *genes for molecular systematic [27]. This suggests that *rhs *gene repertoires may be highly dynamic, but the true scale of *rhs *diversity, their ubiquity in enteric genomes and their structural and evolutionary dynamics remain unexplored. Here we present a detailed analysis of *rhs *elements in the Enterobacteriaceae, which has three objectives: i) to reveal the scale of *rhs *diversity across enteric bacteria and provide an evolutionary systematic classification; ii) to compare and contrast the *rhs *repertoires between strains, species and genera to characterise the evolutionary dynamics of *rhs *diversification; and iii) provide a mechanistic model of how *rhs *diversity is gained and lost by bacterial genomes. In achieving these objectives, we discount any role for *rhs *elements in chromosomal rearrangement, we show that *hcp *and *vgr *are not present in all *rhs *and by studying *rhs *phylogeny and evolution, we propose a novel mechanism for diversification of these genes through C-terminal displacement.

## Results

### *Rhs *loci are not constituents of specialized 'accessory elements'

From analysis of hundreds of completed bacterial genome sequences, there are many examples of large chromosomal rearrangement mediated via bacteriophage, IS elements and rRNA operons [14,28,29]. However, there is not one single published report of chromosomal rearrangement mediated via *rhs *elements and hence it is clear that these loci are not 'rearrangement hot spots' as originally defined by Hill and co-workers [26]. This lack of support for a function of these genetic elements in chromosomal rearrangements illustrates the artefactual nature of the original phenotype in which these loci were discovered and provokes a need to assess what the function of these 'elements' are in bacterial cells; moving away from considering *rhs *elements as *cis*-acting 'DNA elements' or 'accessory elements' as they are widely annotated, to actually consider them as simple genes that encode a large protein, defined by Hill and co-workers as the Rhs 'core' protein [21]. Hence, when we refer to *rhs *we will use the term *rhs *gene and not *rhs *element to reinforce that fact that we consider these essentially to be a normal protein encoding genes.

### The canonical primary structure of Rhs proteins

**Figure 1:**
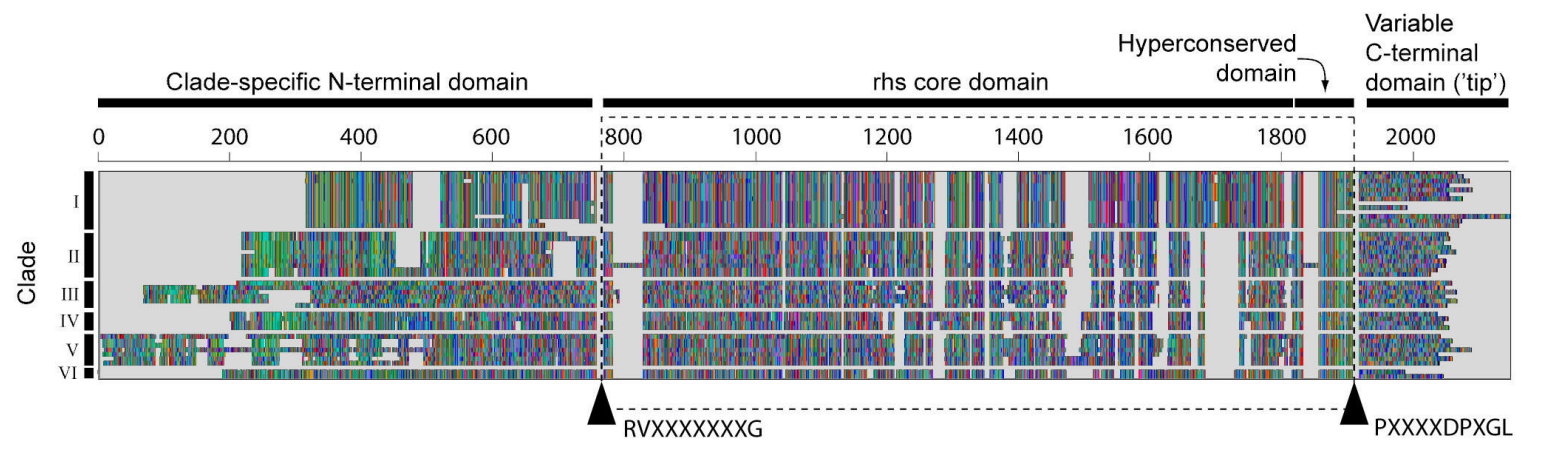
**A multiple alignment of *rhs *protein sequences from across the Enterobactericaeae**. Scale in amino acid residues. The alignment is divided into four: 'clade-specific' N-terminal domain, core domain, (including a hyperconserved domain) and variable C-terminal domain. The region used in phylogenetic analyses is bordered by a dotted line and conserved amino acid motifs. Clade structure (refer to Figure 2) is shown at left.

From our comprehensive sample of enterobacterial *rhs *genes, we have redefined the primary protein structure originally described by Hill [21,26], given that the family comprises greater structural variety than previously appreciated. Figure 1 shows that each Rhs protein consists of four distinct domains: i) a 'clade-specific' N-terminal domain (365-695 amino acids) that is conserved within, but not between, subfamilies of *rhs*; ii) a 'core domain' (776-888 amino acids) that is flanked by conserved motifs. The core varies greatly across all Rhs proteins, but can be aligned due to the conserved secondary structure and, in particular, the series of G residues (often followed by R) found at intervals of 9-11 amino acids, recognised previously by Hill [21]; iii) a very conserved 61 amino acid motif, ending in DPXG-(18)-DPXG and shared by all Rhs proteins, which defines the 3' boundary of the core domain; and iv) an apparently variable C-terminal 'tip' of between 21 and 168 amino acids, which is non-homologous between subfamilies, indeed, often entirely different within subfamilies, individual species and loci. Our sample also confirms that *rhs *genes are often found immediately downstream of a *vgrS *locus, although this is not true in *E. coli *(see below). Like Hill, we observe that *rhs *genes are generally followed by a GC-poor downstream region containing additional, fragmentary *rhs *core and tip sequences. The dynamics of these 'dissociated' tips are analyzed below, after first addressing the global diversity and evolutionary history of *rhs *core sequences.

### Phylogenetic systematics

Our data sample comprised 67 completed and largely published enterobacterial genome sequences, representing 13 different genera and 33 species; in total, 81 *rhs *gene sequences were compared, 6 of which were partial. The *rhs *gene complements of each species are shown in Table 1 and range from 6 or 7 genes in some *E. coli *strains to no *rhs *genes at all in *Sodalis glossinidius *and *Klebsiella *spp. Overall, the total *rhs *complement is highly variable, even within genera, suggesting that *rhs *genes can be gained and lost easily. Multiple alignment of translated nucleotide sequences was possible within the redefined core domain (i.e., N- and C-terminal domains were not used), producing an 1167 character (3501 bp) data set with numerous gaps.

**Table 1:** Enterobacteriaceae species and strains represented in this study, with their *Rhs *gene complement (excluding pseudogenes and gene relics).

Genus	Species	Strain	GenBank Accession Number †	Rhs complement by clade:							
				I	II	III	IV	V	VI	Total	
*Citrobacter*	*rodentium*	ICC168	(WTSI)	5	0	0	0	1	0	**6**	*
*Citrobacter*	*koseri*	BAA-895	CP000822	0	0	0	0	0	0	**0**	
*Citrobacter*	*youngae*	ATCC 29220	NZ_ABWL00000000	2	0	0	1	0	0	**3**	*
*Dickeya*	*dadantii*	3937	(ASAP)	0	0	0	2	1	0	**3**	*
*Enterobacter*	*sakazakii*	ATCC-BAA-894	CP000783	0	0	2	0	0	0	**2**	*
*Enterobacter*	*tasmanensis*	Et1/99	NC_010694	0	1	2	0	0	0	**3**	*
*Enterobacter*	*sp.*	638	CP000653	0	0	0	0	0	0	**0**	
*Erwinia*	*amylovora*	Ea273	(WTSI)	0	3	1	0	0	0	**4**	*
*Erwinia*	*carotovora*	ATCC BAA-672	BX950851	0	0	0	2	1	0	**3**	*
*Escherichia*	*albertii*	TW07627	NZ_ABKX00000000	1	0	0	0	1	0	**2**	*
*Escherichia*	*coli*	536	NC_008253	0	0	0	0	0	0	**0**	
*Escherichia*	*coli*	APEC_01	NC_008563	0	0	0	0	0	0	**0**	
*Escherichia*	*coli*	ATCC8739	NC_010468	5	0	0	1	0	0	**6**	*
*Escherichia*	*coli*	CFT073	NC_004431	0	0	0	0	0	0	**0**	
*Escherichia*	*coli*	E24377A	NC_009801	6	0	0	1	0	0	**7**	
*Escherichia*	*coli*	EC0127_H6_E2348.69	NC_011601	0	0	0	0	0	0	**0**	
*Escherichia*	*coli*	EC0157_EC4115	NC_011353	6	0	0	1	0	0	**7**	
*Escherichia*	*coli*	EC0157_H7_EDL933	AE005174	6	0	0	1	0	0	**7**	
*Escherichia*	*coli*	EC0157_H7_Sakai	NC_002695	6	0	0	1	0	0	**7**	*
*Escherichia*	*coli*	HS	NC_009800	3	0	0	0	0	0	**3**	
*Escherichia*	*coli*	K12_MG1655	NC_010473	4	0	0	0	0	0	**4**	*
*Escherichia*	*coli*	SE11	NC_011415	6	0	0	1	0	0	**7**	
*Escherichia*	*coli*	SMS.3.5	NC_010498	0	0	0	0	0	0	**0**	
*Escherichia*	*coli*	UTI89	NC_007946	0	0	0	0	0	0	**0**	
*Escherichia*	*fergusonii*	ATCC 35469T	CU928158	0	0	1	0	0	0	**1**	*
*Klebsiella*	*pneumoniae*	MGH 78578	CP000647	0	0	0	0	0	0	**0**	
*Klebsiella*	*sp.*	342	CP000964	0	0	0	0	0	0	**0**	
*Photorhabdus*	*luminescens*	laumondii	BX470251	0	1	0	1	1	0	**3**	*
*Photorhabdus*	*asymbiotica*	ATCC43949	FM162591	0	1	0	1	1	0	**3**	*
*Proteus*	*mirabilis*	H14320	AM942759	0	1	0	0	1	0	**2**	*
*Salmonella*	*enterica*	Agona_SL483	NC_011149	1	0	0	0	0	0	**1**	*
*Salmonella*	*enterica*	Gallinarum_287.91	AM933173	1	1	0	0	0	0	**2**	*
*Salmonella*	*enterica*	Cholerasuis	AE017220	0	0	1	0	0	0	**1**	*
*Salmonella*	*enterica*	Dublin_CT_02021853	NC_011205	0	0	1	0	0	0	**1**	
*Salmonella*	*enterica*	Heidelberg_SL476	NC_011083	0	0	1	0	0	0	**1**	
*Salmonella*	*enterica*	Paratyphi_A_AKU_12601	CP000026	0	0	1	0	0	0	**1**	*
*Salmonella*	*enterica*	PT4_P125109	NC_011294	0	0	1	0	0	0	**1**	*
*Salmonella*	*enterica*	Schwarzengrund_CVM19633	NC_011094	0	1	0	0	0	0	**1**	
*Salmonella*	*enterica*	*typhimurium *LT2	AE006468	0	1	0	0	0	0	**1**	*
*Salmonella*	*enterica*	*typhi *CT18	AL513382	0	1	1	0	0	0	**2**	*
*Salmonella*	*enterica*	*typhi *Ty2	AE014613	0	1	0	0	0	0	**1**	*
*Salmonella*	*enterica*	Newport_SL254	NC_011080	0	1	0	0	0	0	**1**	
*Salmonella*	*enterica*	Paratyphi_B_SPB7	NC_010102	0	0	0	0	0	0	**0**	
*Salmonella*	*enterica*	*arizonae *62_z4.z23	NC_010067	0	0	0	0	0	0	**0**	
*Salmonella*	*bongori*	ATCC 43975	(WTSI)	1	0	0	0	1	0	**2**	*
*Serratia*	*proteamaculans*	568	CP000826	0	0	0	0	0	0	**0**	
*Serratia*	*marcescens*	Db11	(WTSI)	0	1	1	0	0	0	**2**	*
*Shigella*	*sonnei*	Ss046	CP000038	3	0	0	1	0	0	**4**	*
*Shigella*	*boydii*	CDC_3083.94	NC_010658	3	0	0	0	0	0	**3**	
*Shigella*	*boydii*	Sb227	NC_007613	3	0	0	0	0	0	**3**	*
*Shigella*	*dysenteriae*	Sd197	NC_007606	3	0	0	0	0	0	**3**	*
*Shigella*	*flexneri*	2a_2457T	AE014073	0	0	1	0	0	0	**1**	
*Shigella*	*flexneri*	5_8401	CP000266	0	0	1	0	0	0	**1**	*
*Sodalis*	*glossinidius*	*morsitans*	NC_007712	0	0	0	0	0	0	**0**	
*Yersinia*	*pestis*	C092	AL590842	0	1	0	0	1	1	**3**	*
*Yersinia*	*pseudotuberculosis*	IP32953	BX936398	0	1	0	0	1	2	**4**	*
*Yersinia*	*pseudotuberculosis*	YAPI pathogenicity island	CAF28563	0	1	0	0	0	0	**1**	*
*Yersinia*	*enterocolitica*	8081	AM286415	0	0	0	0	0	0	**0**	
*Yersinia*	*bercovieri*	ATCC43970	NZ_AALC00000000	0	0	0	0	0	0	**0**	
*Yersinia*	*frederiksenii*	ATCC33641	NZ_AALE00000000	0	0	0	0	0	0	**0**	
*Yersinia*	*intermedia*	ATCC29909	NZ_AALF00000000	0	0	0	0	0	0	**0**	
*Yersinia*	*mollaretti*	ATCC43969	NZ_AALD00000000	0	0	0	0	0	0	**0**	

Note: An asterisk * signifies that a given genome sequence was used in the global phylogenetic analysis (see Methods).

All sequences are deposited in GenBank, either in finished or draft condition, except for those sequences marked with a cross †; these are publicly available from the Wellcome Trust Sanger Institute (WTSI) or University of Wisconsin-Madison (ASAP).

**Figure 2:**
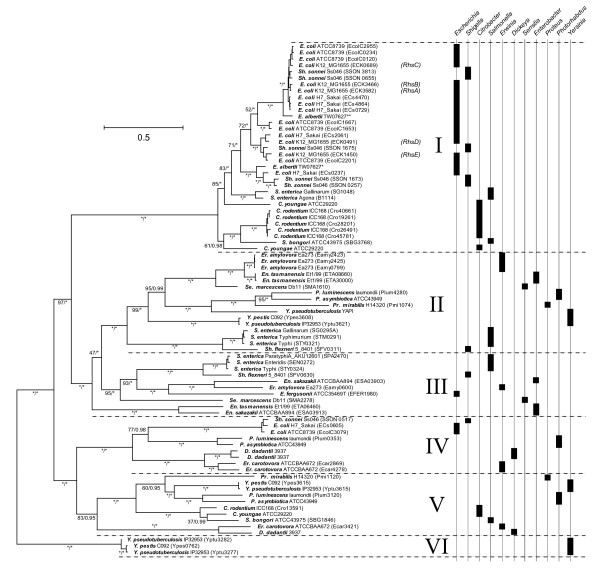
**Maximum likelihood phylogeny showing global *rhs *genetic diversity**. The ML phylogram was estimated from a multiple alignment of 81 *rhs *core domain nucleotide sequences from 11 genera, using a GTR+Γ model. Scale is in substitutions/site. The topology is concordant with alternative trees estimated from protein sequence alignments, and with Bayesian phylogenies. Node support is provided by non-parametric bootstrap values/Bayesian posterior probabilities; an asterisk * denotes values of 100/1.00 respectively. Each terminal node is labelled with the species (in bold), strain and locus tag, where available. Existing *rhs *aliases are shown in red next to *E. coli *K12 *rhs *sequences. The tree is subdivided into six clades and the phylogenetic distribution of each bacterial genus is shown on the right.

Phylogenetic analysis produced a robust and reliable estimate under various conditions. The ML phylogram for nucleotide sequences is shown in Figure 2; this topology was also recovered from Bayesian analysis of nucleotide sequences, as well as analysis of amino acid sequences (not shown). All trees support six clades (labelled I to VI) with robust bootstrap values (> 95), reflecting distinct primary structures belonging to each clade. This cladistic pattern corresponds with the highly distinct N-terminal sequences (which were not used in phylogenetic estimation), providing independent validation of the relationships. We confirmed that the phylogeny was not affected by base composition or codon usage bias; *rhs *from the same clade but different genomes had dissimilar base composition and codon usage scores, while *rhs *from different clades but the same genomes, for instance *rhs *from *Yersinia *spp., had similar codon usage scores (data not shown).

Of the six clades, I and VI are limited in taxonomic distribution. Clade I is primarily associated with *E. coli/Shigella *spp. in which it has been substantially expanded through gene duplication. It is also found in *C. rodentium, S. bongori*, and some strains of *S. enterica*. Clade VI is restricted to *Yersinia *spp. Clades II-IV are widespread; each has representatives in genomes from across the Enterobacteriaceae, indicating that they were present in the common ancestor. However, the distribution is also punctate, i.e., although widespread, Clades II-IV are not universal. Thus, gene loss, and perhaps recovery, has occurred frequently as the Enterobacteriaceae have diversified. Having established the global diversity of all *rhs *genes, we focussed our analysis on two particularly well sampled taxa (*Salmonella enterica *and the *E. coli/Shigella *complex) to understand what regulates *rhs *diversity on a local scale, i.e., among strains.

### *Rhs *loci in *Salmonella enterica*

From the global analysis of Table 1 and Figure 2, it is clear that most *S. enterica *strains possess a single *rhs *gene, belonging to either Clade II or III. More rarely, *S. enterica rhs *repertoire includes a Clade I gene (specifically strains Agona and Gallinarum). In the phylogeny, these genes fall definitely outside the bulk of *E. coli *Class I genes, suggesting that they are true orthologs and not the result of LGT. Occasionally, as with Paratyphi *A *and Arizonae strains, gene loss has entirely removed all *rhs *genes. A closer examination of *S. enterica*, described in Table 2, shows that both taxon and position define *rhs *repertoire. All *rhs *loci are found on the mobile genetic elements SPI6 and ROD9, the former being the most common. The SPI6 locus always contains a Clade II or III *rhs*, the exception being strain Typhimurium, which has both Clade II and III genes arranged in tandem. The ROD9 locus contains a Clade I *rhs *and is found only in Agona and Gallinarum strains, although these strains display evidence of deleted *rhs *genes at the SPI6 locus in addition. In contrast to *S. enterica*, *S. bongori *does not conform to these rules; it possesses two *rhs *genes that are Clade I- and V-types respectively; and these loci are not found on mobile elements. Instead, the unrelated Clade I- and V genes are each closely related to counterparts in *C. rodentium *and *C. youngae*, strongly indicating that they have been acquired through LGT from *Citrobacter*, or some third party.

**Table 2:** *Rhs *loci on two genomic islands in *Salmonella enterica *strains.

Strain	Position:			
	*SPI6*		*ROD9*	
		Clade		Clade
Agona_SL483	Y*	-	Y	I
Gallinarum_287.91	Y*	II	Y	I
Cholerasuis	Y	III	N	-
Dublin_CT_02021853	Y	III	N	-
Heidelberg_SL476	Y	III	N	-
Paratyphi_A_AKU_12601	Y	III	N	-
Enteridis P125109	Y	III	N	-
Schwarzengrund_CVM19633	Y	II	N	-
Typhimurium_LT2	Y	II	N	-
Typhi_CT18	Y	II and III	N	-
Typhi_Ty2	Y	II	N	-
Newport_SL254	Y	II	N	-
Paratyphi_B_SPB7	N	-	N	-
Arizonae	N	-	N	-

An asterisk * signifies that the SPI6 locus is represented by a pseudogene or gene relic.

### *Rhs *loci in *Escherichia coli*

The situation in *Salmonella *contrasts starkly with that in *E. coli *and *Shigella*. Instead of a single *rhs *gene housed within a mobile element, there are multiple *rhs *loci distributed throughout the genome. We saw in the global analysis that these loci largely belong to Clade I. In the phylogeny, *E. coli *Clade I genes have short branch lengths and are monophyletic, i.e., they are more related to each other than anything else. These close relationships show that diversification has been relatively recent; indeed, strain-specific loci and ubiquitous variation in repertoire among strains suggest that *rhs *expansion in *E. coli *is an on-going process. However, Clade I *rhs *exist in *S. enterica *and *C. rodentium*, showing that the clade predates *E. coli*, although it probably does not predate the Enterobacteriaceae since it is not represented widely. *E. fergusonii*, which is a true congener of *E. coli*, lacks Clade I *rhs *but has Clade III. Given that *Salmonella *possesses Clades I-III, this indicates that the ancestral *Escherichia *had a more diverse repertoire than *E. coli*. In summary, the *E. coli rhs *repertoire represents a contraction of ancestral state, coupled with a substantial and species-specific evolutionary expansion of Clade I *rhs*.

**Figure 3:**
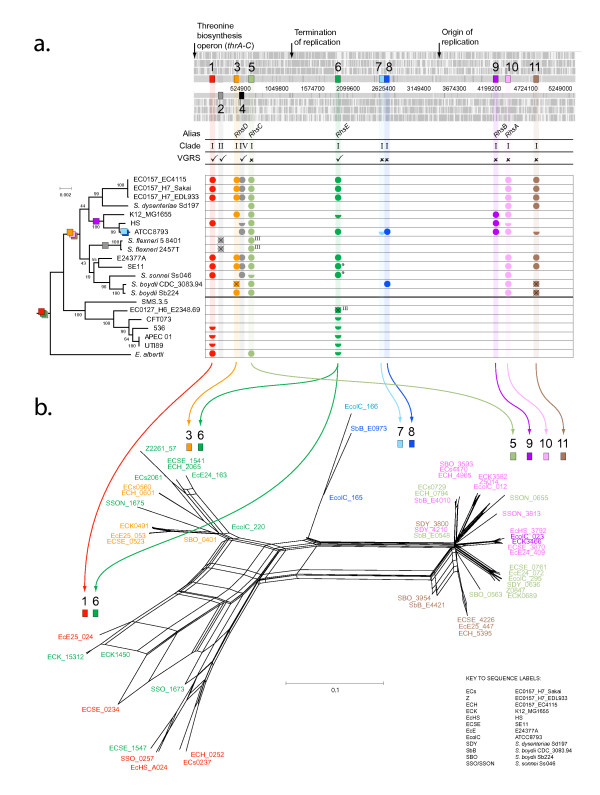
**Comparative genomics and phylogenetics of *rhs *genes in *E. coli/Shigella *spp**. **a**. *Rhs *loci are found at 11 unique positions in *E. coli*. These are marked along the K12_MG1655 genome (scale in base-pairs, beginning at the thr operon). For each locus the following are noted: the existing gene name ('alias') where available, the clade to which it belongs (see Figure 2), the presence or absence of a contiguous vgrs gene, and its phylogenetic distribution across all strains (present: solid circle, pseudogene: crossed through, relic: half circle, or otherwise absent). Note that two sequences labelled 'III' belong to Clade III, rather than Clade I. The ML phylogeny shown at left was estimated from MLST concatenated sequence (see Methods), and is labelled with bootstrap values. Coloured boxes denote the inferred origins of rhs loci. **b**. A phylogenetic network estimated from HKY distances using a Neighbour-Net algorithm. Sequence labels are shaded by locus, as in **a**. A key is provided that relates strain names to sequence codes. Clades are linked to their corresponding positions by arrows.

In total, there are 11 unique *rhs *loci among the 20 strains included here, which are defined in Table 3 by their conserved flanking genes and superimposed on the K12 genome in Figure 3a. Obviously, not all strains possess genes at all positions, although some (i.e., 1, 3, 5, 6 and 10) are better attended than others. Most positions contain Clade I *rhs*, but position 4 contains a Clade IV *rhs*, (as noted in the global analysis) and position 2 contains a Clade II gene that is specific to *Sh. flexneri*. The two *rhs *genes of *Sh. flexneri *are an anomaly: the first is Clade II-type and found uniquely at position 2, the second is Clade III-type and found at position 5 (which hosts Clade I *rhs *in all other strains). Hence, *Sh. flexneri *seems to have a *S. enterica*-like repertoire in an *E. coli*-like setting; the phylogeny corroborates this, since both genes cluster tightly with their *S. enterica *homologs. This indicates that *Sh. flexneri *has replaced its ancestral (*E. coli*-like) *rhs *through LGT from *S. enterica*.

**Table 3:** Unique genomic positions of conserved *Rhs *loci in *E. coli *and *Shigella *spp.

Locus	Position (bp)	Strand	Upstream gene	ECK#	Downstream gene	ECK#
1	240500	F	DNApol III epsilon subunit, dnaQ	0215	acyl-CoA dehydrogenase, fadE	0222
2	318500	R	acyl-CoA dehydrogenase, fadE	0222	oxidoreductase, ykgE	0305
3	523600	F	ABC transporter, ybbA	0489	selenouridine synthase, ybbB	0496
4	591800	R	N4 receptor, nfrB	0561	histidine kinase, cusS	0562
5	730400	F	ATPase, kdpA	0686	deoxyribodipyrimidine photolyase, phr	0697
6	1526800	F	putative transferase, yncG	2058	oxalocrotonate tautomerase, pptA	2064
7	2054000	F	MATE efflux protein, yee0	1980	phosphoribosyl transferase, cobT	1986
8	2041000	F	Cytochrome b561, yodB	1970	transcription factor, yeeJ	1974
9	3620000	F	transcriptional repressor, nikR	3465	ABC transporter, yhhJ	3470
10	3760000	F	Glutathione S-transferase, yibF	3581	Putative membrane protein, yibH	3586
11	4125000	F	Peptidoglycan peptidase, yiiX	3937	Primosome assembly protein, priA	3935

Note: Positions are given relative to the K12_MG1655 genome sequence, beginning at the *thr *operon.

An asterisk * denotes that *Sh. flexneri *has a Clade III *Rhs *at this position.

In Figure 3a, *rhs *repertoires are mapped onto a phylogeny of *E. coli/Shigella *strains, with *E. albertii *as an outgroup. Several features emerge from this analysis: i) one group of pathogenic strains (below the heavy line) lack the evolutionary expansion of Clade I *rhs *and, furthermore, have only vestiges of positions 1 and 6 (showing that these have been deactivated); ii) gene loss has also affected other strains creating widespread genomic variation, even between related strains, for instance among K12, HS and ATCC8739; iii) position 8 occurs in an evolutionary labile region and is only conserved in *Sh. boydii *CDC and ATCC8739, which is anomalous since these strains are not closely related. It suggests that one or other strain has acquired the locus through LGT; and iv) *E. albertii *possesses complete genes at positions 1 and 5, as well as a relic at position 6, indicating that these loci are oldest, predating the origin of *E. coli*. Hence, we have an evolutionary hypothesis of the Clade I *rhs *expansion. The ancestral *E. coli *had *rhs *at positions 1, 5 and 6, which have become non-functional in some strains. Position 3 and positions 10 and 11 evolved after the split with the pathogenic strains, through duplication of position 6 and 5 respectively; the remaining loci evolved uniquely in the K12/HS/ATCC8739 lineage (position 9); *Sh. flexneri *(position 2) and ATCC8739 (positions 7 and 8).

Having looked in detail at the phylogenetic distribution of Clade I *rhs *loci among strains, we estimated a phylogenetic network to examine the relationships among *rhs *gene sequences, but now including their variable C-termini. The results are presented in Figure 3b and show that the independent gene presence/absence and nucleotide sequence data generally agree. Most importantly perhaps, the network shows that sequences cluster by genomic position, rather than by taxon or randomly. This indicates that recombination between cores at different Clade I loci is infrequent (if any) and, from a pragmatic view, all genes at a given position can be considered orthologous when comparing *E. coli *strains. The relationships between these clusters corroborate the diversification scenario inferred from Figure 3a; *rhs *from positions 1 and 6 are most divergent, consistent with their origin being the earliest of all. The *rhs *genes at position 6 appear in two positions within the network: most sequences cluster with *rhs *from position 3, but sequences from strains K12, SE11 and *Sh. sonnei *cluster with *rhs *at position 1. Interestingly, both types are found at position 6 in *E. coli *SE11 and *Sh. sonnei*.

Finally, although clade I *rhs *core sequences cluster by genomic position, the network shows that *rhs *at different positions can share a common pool of variable C-terminal 'tips'. *Rhs *core sequences at positions 5, 9-11 are almost identical, as seen from the tight clustering at the right-hand end of Figure 3b, and are distinguished mainly by the non-homologous tips attached to otherwise invariant core sequences. It can be seen that the same tip can be attached to *rhs *genes at different clade I loci, for instance, between positions 9 and 10, or 10 and 5. When we inspect the C-terminal tips for all clade I *rhs *in *E. coli *(see Additional File 1), it is clear that tips can be shared even between *rhs *genes with quite dissimilar core sequences, for instance between positions 3 and 9-11. Conversely, a given position can harbour several different tips across all strains. Altogether, a survey of the associated C-terminal tips in clade I *rhs *suggests that tips can be exchanged between loci, and the total diversity of tips is very large. We sought to find further evidence for this among the dissociated tips found downstream of core sequences.

### Displacement of C-terminal tips

**Figure 4:**
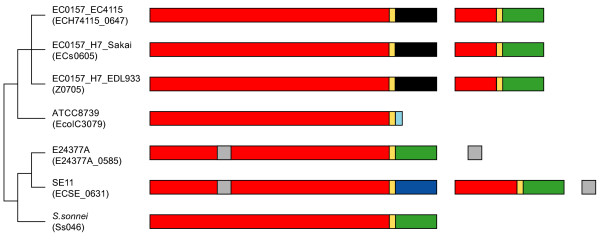
**Arrangement of associated and dissociated C-terminal tips in *Serratia marcescens***. The chromosome sequence is shown in grey, with gene models and G+C content shown plotted above. The regions between dotted lines correspond to *rhs *genes and fragments (core domain: red; hyperconserved domain: yellow; variable C-terminal: various). Scale in base-pairs.

As detailed above the core regions of *rhs *genes are followed by an apparently exchangeable tip, giving the impression of hypervariability. This predicts that there must be a large pool of alternative tips available for exchange. Using the clade II *rhs *locus (SMA1610) in *Serratia marcescens *strain Db11 as a model, Figure 4 shows how the region encoding the core displays a significantly higher G+C content than that encoding the C-terminal tip (67.7% compared with 46.4%; Wang et al. 1998). In many instances this low G+C trough extends beyond the *rhs *gene and, in the case of *Se. marcescens*, > 7 kb downstream. In *Se. marcescens *this region includes three *rhs *gene fragments (SMA1612, SMA1614 and SMA1616) all consisting of a partial core of varying lengths, a full core repeat and recognisable C-terminal tip. Thus far these fragments have been annotated and published as relics of *rhs *genes that have been lost by deletion over time [14,30]. When the core regions of the *Se. marcescens rhs *gene fragments were compared to the core regions of all the *rhs *genes within its genome they were found to be almost identical to the core region of the *rhs *gene located immediately upstream (91.6% amino acid identity), appearing as a series of large direct repeats of varying length. In contrast, the *rhs *fragments each carry an entirely dissimilar C-terminal tip. This combination of invariant core sequences and non-homologous tips within a given locus is consistent with the analysis of clade I *rhs *tips in *E. coli *above. Only one of the *Se. marcescens rhs *fragments (SMA1612) possesses an appropriate translational start site suggesting that they are likely to be functionally silent. Consistent with this, two of the *Se. marcescens rhs *gene fragments (including SMA1612) have further degenerated carrying frameshift mutations and/or premature stop codons (Figure 4).

**Figure 5:**
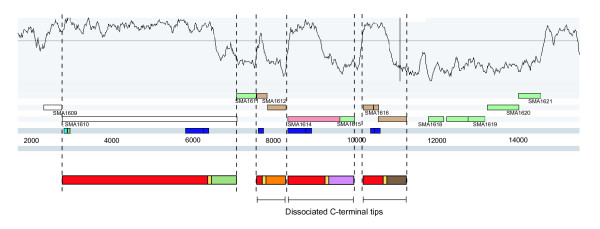
**Comparison of C-terminal tip types in *E. coli*, at position 4 (clade IV rhs)**. The phylogenetic relationships of seven *E. coli *strains are shown at left. Complete and fragmentary *rhs *genes at position 4 in each strain are shaded as in Figure 4. Two strains possess a downstream core domain fragment without any obvious associated tip; the fragment and its corresponding location in the core domain are shaded grey.

*E. coli *clade I loci are unusual in that dissociated tips are generally absent downstream. However, the sole clade IV locus (position 4) in *E. coli *does conform to the pattern described in Figure 4. Therefore, we compared both associated and dissociated tips across seven *E. coli *strains possessing a clade IV *rhs*, which are illustrated in Figure 5. Since we have multiple isolates of the same species sharing the same locus we can add a temporal dimension to the static observations made in *Se. marcescens*. In this instance it is evident that once-attached C-terminal tips have been displaced by the insertion of new tips onto the *rhs *gene because we see a shared, (and presumably ancestral), tip (shaded green) displaced by two different tips independently. As with *Se. marcescens*, when comparing associated and dissociated sequences for each *E. coli *strain, although the C-terminal tips were distinct within a locus, the core regions were always identical. Hence, the apparent variability of C-termini in *rhs *seems to be generated by the insertion of exogenous sequences, resulting in the displacement of the incumbent tip, which then becomes 'dissociated'. Since the process is slow enough for us to observe orthologous tip types across different *E. coli *strains, variability is clearly not generated by rapid sequence divergence; the alternative is that a very large pool of tips has evolved over a long period, in fact, that the tips are well conserved. In support of this, we found that the 'blue' tip in Figure 5 (ECSE_0631) was highly related (74% amino acid identity) to a dissociated tip in *Er. carotovora *(ECA4293). This tip is also located downstream of a clade IV *rhs *gene (ECA4278).

To confirm the general pattern that C-terminal tips are not recently evolved and hypervariable, we compared each associated C-terminal tip from every *rhs *gene in our dataset with the UNIPROT database. This showed that many clade II-V tips have very good matches (70-95% amino acid identity, data not shown) to intact and fragmentary *rhs *in other Enterobacteriaceae, and indeed, in other eubacterial families besides. Where the affinity of the tip could be determined, the core sequences of the query and matched tips belonged to the same clade. C-terminal sequences from clade I and VI *rhs *did not have widespread matches, consistent with the restricted taxonomic distribution of their core sequences (see Figure 2). In summary, our data show that a large pool of structurally conserved C-terminal domains are exchanged between *rhs *genes, through a process of insertion and displacement, but that exchange is limited to members of the same clade.

## Discussion

Our understanding of the *rhs *gene family has been hampered by a very incomplete knowledge of global sequence diversity, due in part to the characterisation of *rhs *in *E. coli *K12, which, as should now be clear, has a relatively meagre and unrepresentative *rhs *repertoire. In fact, *rhs *genes are much more diverse than previously appreciated and comprise six structurally and phylogenetically distinct lineages within the Enterobacteriaceae. The taxonomic distribution of these distinct *rhs *types is punctate, reflecting frequent and independent gene gains and losses in different genera, as well as occasional LGT. When we look closer within particular species, inter-strain variation exposes some of the mechanisms responsible. In *S. enterica*, *rhs *are restricted to mobile elements and are limited to a single functional copy through differential deletion. In *E. coli*, there has been a major expansion in Clade I *rhs *through transposition to novel loci, and loss of other clades relative to other *Escherichia *spp. Comparison of C-terminal tips and dissociated fragments shows that while C-termini vary greatly within a locus, each distinct sequence is conserved in related strains, indeed in other species and genera. Hence, we must conclude that they are structurally conserved rather than hypervariable, that is, C-terminal variability is facilitated by dynamic substitution from a theoretically large pool of structurally diverse, (but evolutionarily old), C-terminal sequences rather than rapid divergence of static sequences under selection. These auxiliary C-termini are not all resident on the same chromosome and so must exist episomally and, since related strains can often have the same arrangement of dissociated *rhs *fragments, displacement of one C-terminus by another proceeds relatively slowly.

The structural features of *rhs *loci, the combination of conserved and variable domains coinciding with distinct GC signatures and the tandem repetition of gene fragments downstream of an intact gene copy, have analogues in other organisms. In *Neisseria meningitidis*, the *mafB *genes occupy three distinct loci and each is arranged in this way; a full-length gene copy is followed by C-terminal fragments of variable length and low GC-content, but flanked by conserved domains [31,32]. Comparative genomics indicates that a given C-terminal tip can be associated with a complete *maf *gene in some strains, and found unattached downstream in others, mirroring the evidence presented here for *rhs *[32]. There is no evidence that *rhs *and *mafB *are homologous, and so their structurally analogy may reflect a convergence enforced by common mechanistic constraints.

The structure of *rhs *genes and their downstream silent tips has superficial similarities with gene variation mechanisms in several unrelated bacterial pathogens including the pilus antigenic variation system seen in *Neisseria gonorrhoeae *[33-35], the haemagglutinin in *Mycoplasma synoviae *[36], and antigenic variation in *Borrelia *[37-39]. In these systems variation is introduced into the expressed gene by recombination between itself and one of a number of silent copies of that gene located either downstream or elsewhere in the genome. All gene copies are composed of constant and variable regions where the latter show little conservation but are flanked by conserved sequences that facilitate recombination. As stated above, *Rhs *genes are not hypervariable and are not truly analogous to systems of antigenic variation, but recombination may be similarly employed to introduce structural variability. Our observation of C-terminal displacement is also superficially reminiscent of the 'terminal reassortment' process that might create novel type III secretion systems through the generation of sequence mosaics [40]. However, this is presented as a largely unregulated process of highly promiscuous recombination between unrelated genes, while our observations suggest a process tightly regulated by structural homology and, consequently, with much less scope for introducing novelty.

**Figure 6:**
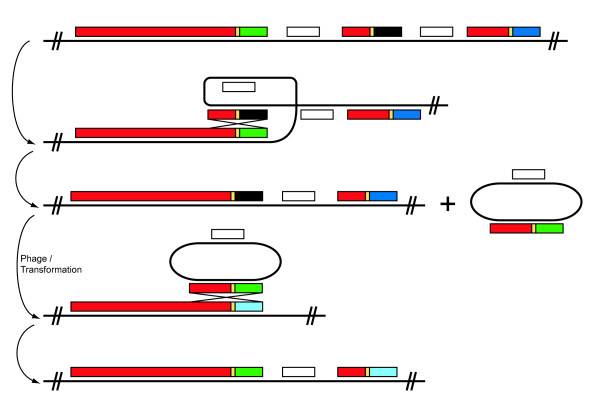
**A hypothetical model of C-terminal tip displacement**. Homologous recombination between the conserved core sequence within the downstream unattached tip and either the core region of the *rhs *gene or that of another unattached tip. This event would result in the production of a recombination proficient episomal circle carrying a conserved core region, of varying lengths, which is attached to a variable C-terminal tip and any intervening genes. After transfer to a second bacterial isolate homologous recombination would be required between the highly conserved core regions on the chromosomal *rhs *gene and identical sequences located on the episomal circle carrying the unattached tip. This second single cross-over event of a circular intermediate is required to explain how an attached tip can be displaced by a new tip without deleting or entirely replacing the old tip, but simply shunting it to a silent position downstream of the intact *rhs *gene.

In proposing a mechanism for how within-genome *rhs *sequence variability is generated, we need to explain the principal observation that *rhs *fragments consisting of core domain sequence and variable C-termini are found dissociated downstream of intact genes, often in long strings. Our essential contention is that this reflects previous displacement events by 'incoming' tips, but we also need to explain why the core fragments are of variable length and why tips at a given locus only ever contain core sequences of the same clade, (i.e., why are there inherent phylogenetic limits on what can insert). We propose a model that requires two independent recombination events, which is described in Figure 6. First, there is homologous recombination between the conserved core sequence within a dissociated tip and either the core region of the *rhs *gene or that of another unattached tip. This would result in the production of a small episomal circle carrying a conserved core region that is attached to a variable C-terminal tip and any intervening genes. The length of the core fragment would depend on where the recombination breakpoint occurred. Second, after transfer to another bacterial isolate, homologous recombination would be required between the chromosomal *rhs *core and identical sequences located on the episomal circle carrying the unattached tip. The phylogenetic limits of displacement would indicate that very high identity is required between core domain sequences to permit recombination. This second cross-over event involving a circular intermediate is required to explain how an attached tip can be displaced by a new tip without deleting or entirely replacing the old tip, but simply shunting it to a silent position downstream of the intact *rhs *gene.

All circumstantial evidence supports existing tips being repeatedly replaced by non-homologous alternatives, yet there is little evidence that these new tips originate from other locations in the same genome. This means that new sequences must be acquired from other bacteria with related *rhs *elements; or alternative tips could be carried into the genome as cargo on other mobile genetic elements. The former would require that the episomal circle carrying the alternative tip is sufficiently stable so that it could be transferred between bacteria. This could occur by generalised transduction or natural transformation. Precedents for such a stable circular intermediate come from both integron-mediated gene exchange [41], which requires an integrase that is not known to co-occur with *Rhs*, and from the pilus antigenic variation system in *Neisseria*, in which recombination between *pilE *and the silent *pilS *genes does not involve a site-specific integrase but instead utilises the host RecA machinery [42]. Episomal circles carrying pilin tips are sufficiently stable to be used to naturally transform *Neisseria *[35]. With regard to the introduction of new tip by LGT, we have provided evidence that *rhs *genes and alternative tips are frequently carried on genomic islands, such as SPI-6 and ROD9 in *Salmonella*. However, these alternative tips would still need to form stable closed circles to be able to insert at related *rhs *loci.

This study has shown that the *rhs *gene family is ancient and a core component of Enterobacterial genomes, and their structural diversity suggests that they have multiple roles. While C-terminal displacement engenders structural flexibility, which is itself ancient, *rhs *do not show any hypervariability that might indicate they were interacting with host immune systems; indeed, we have shown that pathogenic *E. coli *generally lack *rhs *present in commensal strains. While the biological function of Rhs proteins is still rather unclear, the presence of the repeated motif that is found in other surface proteins like the wall-associated protein WapA from *Bacillus subtilis *[43] and the teneurin family of proteins [23,44,45] suggest a cell surface-associated binding function.

A small number of recent studies have provided potential clues to the functions of Rhs proteins in *E. coli*, although none offer conclusive evidence for function. The most recent and detailed study of RhsA was during an analysis of the mechanism of secretion of group 2 capsular antigens in *E. coli *[46]. RhsA was identified as a likely component of a large hetero-oligomeric capsule biosynthesis/export complex, based on crosslinking to the KpsD protein *in vivo*. In an *rhsA *mutant, levels of the group 2 capsule were reduced in *E. coli *and the KpsD and KpsE proteins were no longer localised at the poles of the cells, suggesting a direction function of RhsA in the assembly and/or functioning of the capsular export pathway. Another study presented evidence that an *rhsA *mutant of *E. coli *O26:H- has a significant colonisation defect in calves compared to the wild-type strains suggesting an important role for RhsA *in vivo *[47]. Finally, a study examining the response of *E. coli *to the biocide polyhexamethylene biguanide (PHMB) reported the increased expression of a number of *rhs *genes in *E. coli *after exposure to PHMB [48]. Despite this rather disparate data, it is clear that the functions of Rhs protein are at the cell surface or cell envelope and their molecular function may well include a role in carbohydrate binding of some form. However, the role of the alternative tip structures and the functional consequences of tip replacement are yet to be elucidated.

## Conclusion

In this study we have shown that, rather than being 'accessory elements' of some kind facilitating rearrangements of bacterial chromosomes, *rhs *genes are an ancient family comprising six distinct lineages in enteric bacteria, most of which predate the origin of the Enterobactericeae itself. Although this venerable heritage suggests that *rhs *genes are a fundamental core component of enterobacterial genomes, we find that they can be gained and lost frequently and over short periods of evolutionary time, resulting in a high turnover. Individual species may very often lack *rhs *entirely, but few genera possess neither *rhs *genes nor evidence of recent loss. The dynamic nature of *rhs *copy number is matched by local variation in the structure of loci. We propose that the long strings of *rhs *genes and gene fragments that have been observed are created by sequential insertion events, whereby existing C-terminal tips are displaced with non-homologous alternatives. Since all attached and dissociated core fragments belong to the same *rhs *clade, as defined systematically, we can state that displacement is fundamentally limited by the phylogenetic distance of subject and donor sequences.

This and other circumstantial evidence causes us to propose a two-stage mechanism in which homologous recombination excises and inserts C-terminal tips attached to core fragments, via a proposed circular intermediate. Should this be so, it will be interesting to consider how such a circular intermediate might be generated in the first instance. Our observation that both attached and dissociated tip sequences are evolutionarily conserved demonstrates that the system of C-terminal displacement is not recent, and that novel *rhs *structures are created through recombination of conserved sequences, rather than rapid mutation or hypervaribility. Accordingly, we hypothesize that a large reservoir of dissociated *rhs *tips exists episomally, (since it is evidently not present on the chromosome), and shared by multiple strains, perhaps even species, at a population level.

## Methods

### Data collection

We assembled a comprehensive sample of *rhs *genes from published or draft released Enterobacterial genome sequences, listed in Table 1. This sample was sufficient to answer global questions about *rhs *diversity, (i.e., concerned with very long periods of time): variation in gene repertoire between and within genera, and the relative contribution of differential gene loss and lateral transfer to the evolution of *rhs *repertoire. And also to answer specific questions concerned with evolutionary dynamics over shorter time periods: *rhs *turnover within bacterial species and the genomic origins of novel *rhs *loci. Only completed genomes were used to better ensure that conclusions regarding gene loss could not be undermined by missing data. *Rhs *genes were identified from genome sequences (irrespective of the quality of annotation) in two ways. First, all known *rhs *genes were placed in a sequence file against which genome sequences were compared using tBLASTn. Any novel sequences were then added to the file. Second, where possible, a genome was compared with a related sequence using the Artemis Comparison Tool (ACT; [49]) to check known *rhs *(or *VgrS*) positions for *rhs *genes or gene relics. Dissociated C-terminal tips were generally identified in the downstream regions of *rhs *loci by the presence of a hyperconserved region, identical to that found in the upstream *rhs *copy. The boundries of the dissociated tip could be defined by an associated peak in GC content and a region of sequence homology with the *rhs *core.

### Multiple sequence alignment

All sequence alignment was carried out on translated nucleotide sequences, which were then back-translated for downstream analysis; this ensured that codon structure was maintained. Initial automated alignments using ClustalW [50] showed that N-terminal domains did not align across all *rhs*, although they did align within clusters (subsequently shown to be clades). C-terminal domains also did not align, even among otherwise well related sequences. Thus, N- and C-terminal regions were removed from the alignment. The remaining 'core' sequence was manually aligned beginning at the 3' end where a hyperconserved region can be found. Working in a 5' direction from this point of unambiguous alignment, orientation of the *rhs *sequences was dependent on conserved cysteine residues that occur at regular intervals, as noted previously [21]. It was assumed that these reflect an underlying secondary structure that is conserved and allows the sequences to be aligned on a positional basis. Much of the sequence between conserved residues is very divergent and not obviously homologous.

### Global phylogenetic analysis

We first carried out a phylogenetic analysis of global *rhs *diversity across the Enterobacteriaceae. The objective was to provide a systematic description of total *rhs *diversity and so gain an insight into variability in *rhs *repertoire between genera. To do this, we removed some congeneric sequences that, if left in, would result in over-sampling of particular lineages, e.g., *E. coli*. Since different *E. coli *strains are effectively representing the same lineage in a broad-scale analysis, removing these 36 sequences did not affect the outcome. Phylogenetic trees were estimated for a 81 sequence data set representing 11 genera using both maximum likelihood (ML) and bayesian inference (BI) methods. ML trees estimated in PHYML [51,52]; optimal model selected using Modeltest [53] for nucleotides and ProtTest [54] for proteins, with rate heterogeneity estimated from the data. All other options were default. 500 non-parametric bootstraps were applied for robustness measures. BI trees were estimated in MrBayes [55,56] using the 'gammarates' option. 2 parallel chains were run for 10,000,000 generations, sampling every 1,000 generations. The first 1,000 generations were discarded as a 'burn-in'. The Potential Scale Reduction Factor (PSRF) in MrBayes approached 1 in all cases, indicating that these conditions were adequate to ensure a stationary distribution of all parameters.

### Comparative *rhs *repertoire in *E. coli/Shigella *spp

After looking at the global diversity of *rhs *sequences, we focused on the relationships among *rhs *genes within well-sampled genera: *E. coli*, *S. enterica *and *Yersinia*. The objective was to observe *rhs *turnover, and C-terminal tip dynamics, on a shorter timescale and in the context of disease phenotype, to get a better idea of the relative importance of gene gain and loss (differential assortment) vs. genetic transfer. To do this we carried out comparative genomic analyses on 14 *S. enterica *strains, noting their *rhs *repertoires in terms of clade (according to the global analysis) and genomic position. We also recorded the *rhs *repertoires of *E. coli/Shigella *species, supplementing the 7 taxa included in the global analysis with a further 13 genome sequences (a total of 60 *rhs*). Such was the profusion of *rhs *in *E. coli *genomes that we decided to formally define *rhs *loci by their genomic position, relative to the K12 genome sequence. While comparing each *E. coli *or *Shigella *sequence to K12, we recorded the presence, absence or partiality of *rhs *at every position where an *rhs *had been observed in *E. coli*, and defining new positions when a gene had not been seen before. Positions were defined by their flanking loci, such that *rhs *genes in different *E. coli *strains can be directly compared and, in future, novel *rhs *can be integrated into the nomenclature.

We mapped the clade I *rhs *repertoires of our *E. coli *strains on to their ML phylogeny, which was estimated (as described above) from a concatenated nucleotide sequence of all seven standard MLST loci, each extracted from their genome sequences. This demonstrated the pattern of gene gain and loss that follows from variation in *rhs *repertoire. At this point, we discarded the non-clade I *rhs *(positions 2 and 4) from our analyses because all informative variation between strains concerned the remaining 9 clade I positions, and all pertinent information on the clade II and IV-type *rhs *in *E. coli *had been previously revealed by the global analysis.

### Phylogenetic analysis of *rhs *in *E. coli/Shigella *spp

To better understand the microevolutionary dynamics regulating clade I *rhs *evolution at this intraspecific scale, we built a phylogenetic network for the *E. coli *and *Shigella *sequences using the Neighbour-Net algorithm in Splitstree v4.0 [57]. This approach has the advantage of presenting a consensus of all possible relationships among sequences, highlighting those 'splits' that are inherently ambiguous, rather than minimizing ambiguity as a phylogenetic tree would do. As subsequent analysis showed, for recombinant sequences with mixed evolutionary histories, this representation is more realistic. As described above, the variable C-terminal tips were removed from the data set when estimating phylogenetic trees because they could not be aligned. This was possible because enough variation was present in the core sequences to resolve the phylogeny. However, in this data set of clade I *rhs *sequences we compared genes from closely related strains and the C-terminus was often the only feature that distinguished one gene from another. Therefore, the variable C-terminal tips were used in estimating the phylogenetic network to both resolve very closely related sequences, and to demonstrate the presence of homologous tips at different loci of the same genome, or non-homologous tips at corresponding loci in different genomes.

## Abbreviations

Rhs: Rearrangement hot-spot; LGT: Lateral Gene Transfer; CDS: Coding sequence;

## Authors' contributions

APJ, GHT and NRT planned the study, collected and analyzed the data and wrote the manuscript. JP provided guidance in interpreting the results and manuscript preparation. All authors have read and approved the manuscript.

## Supplementary Material

Additional file 1 **Supplementary Figure 1. Rhs C-terminal tip variation in *E. coli***. Multiple sequence alignment of variable C-terminal amino acid sequences ('tips'), clustered by homology. All sequences begin with the 3' conserved motif, and extend to the C-terminus. Sequence labels refer to *E. coli *strain and are colour-coded by genomic position, as defined in the text and Figure 3. Click here for file
