# Supplementary material for: Evolutionary diversification of an ancient gene family (rhs) through C-terminal displacement
Source: BMC Genomics. 2009 Dec 7;10:584. doi: 10.1186/1471-2164-10-584 (PMC2935791; doi:10.1186/1471-2164-10-584)
Supplement: Additional file 1 — Supplementary Figure 1. Rhs C-terminal tip variation in E. coli. Multiple sequence alignment of variable C-terminal amino acid sequences ('tips'), clustered by homology. All sequences begin with the 3' conserved motif, and extend to the C-terminus. Sequence labels refer to E. coli strain and are colour-coded by genomic position, as defined in the text and Figure 3. [file 1471-2164-10-584-S1.DOC]

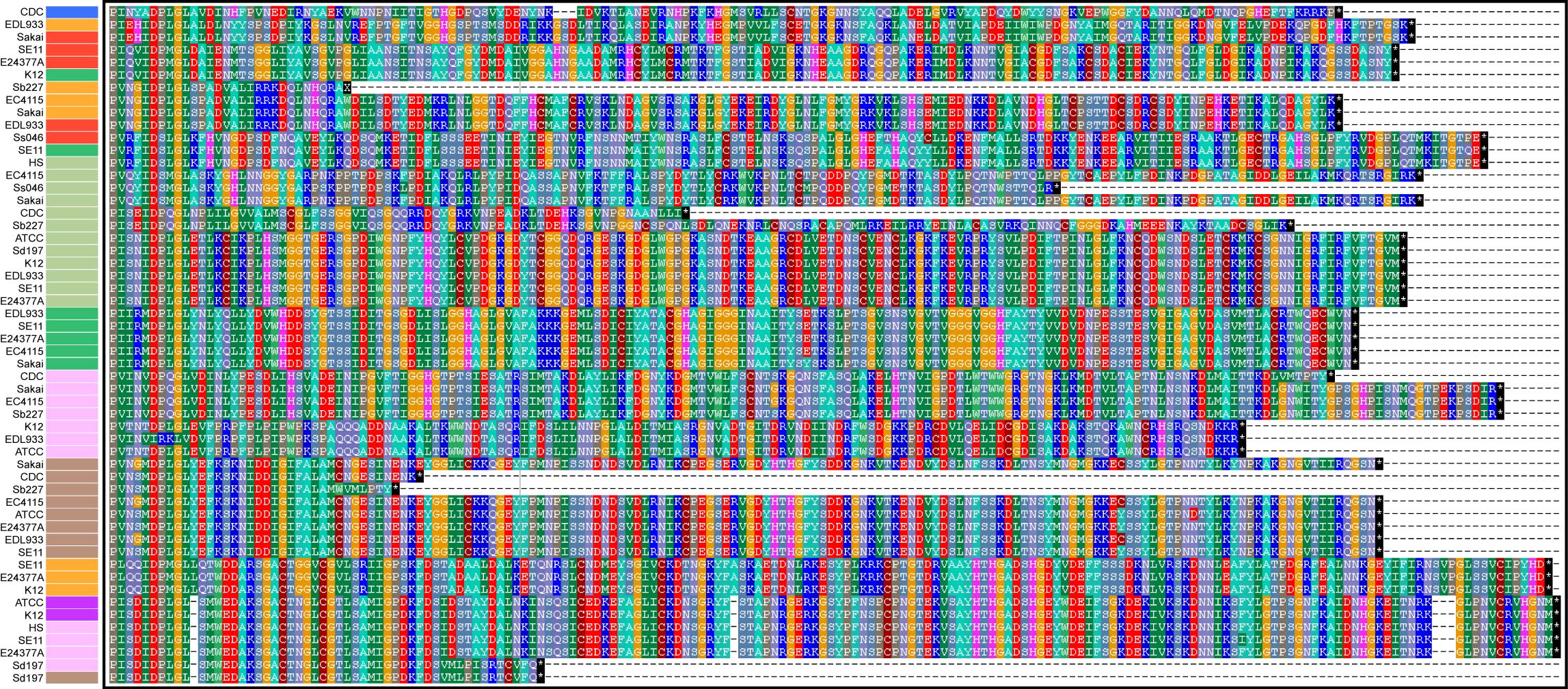


**Supplementary Figure 1**. Multiple sequence alignment of variable C-terminal amino acid sequences (‘tips’), clustered by homology. All sequences begin with the 3’ conserved motif, and extend to the C-terminus. Sequence labels refer to *E. coli* strain and are colour-coded by genomic position, as defined in the text and Figure 3.
